# Domestic Violence Against Women in Nepal: A Systematic Review of Risk Factors

**DOI:** 10.1177/15248380231222230

**Published:** 2024-01-30

**Authors:** Bindu Devkota Sapkota, Padam Simkhada, Dillon Newton, Sara Parker

**Affiliations:** 1 University of Huddersfield, Huddersfield, UK; 2 Liverpool John Moore University, UK

**Keywords:** battered women, domestic violence, cultural contexts, alcohol and drugs, physical abuse, child abuse, violence exposure, anything related to domestic violence

## Abstract

A systematic review was conducted to examine the factors that put women at risk of domestic violence in Nepal. Using the Preferred Reporting Items for Systematic Reviews and Meta-Analyses (PRISMA), PubMed, Cochrane, MEDLINE, CINAHL, and PsycINFO were searched supplemented by searching of the reference list manually. Of the 143 studies identified 24 were included in the final review. Search strategy was developed, and studies were included if they considered female participants (age 15–49 years) in heterosexual relationship, with exposure of different factors and whose outcomes were the magnitude of any form of violence (physical, sexual, and emotional/psychological). The Mixed Methods Appraisal Tool was used to assess the quality of the studies included. The findings are categorized based on the four levels of the ecological framework. At the individual level, the alcohol consumption level of husband, education level of both women and men, women’s age at the time of marriage and childhood exposure to violence were found to be highly prevalent risk factors. At the relationship level, most prevalent risk factors were controlling husband and decision-making capacity of women. At the community level, belonging to underprivileged community or low caste system and living in Terai region were the risk factors. At the societal level, patriarchal belief and norms supporting violence were the risk factors. The complex nature of violence against women in Nepal requires culturally sensitive interventions along with organized efforts from the local and intra government to improve the status of Nepalese women at all levels of the ecological framework.

## Introduction

There is a conflicting view surrounding the terminology used to define domestic violence (DV) (Dixon and Graham-Kevan, 2011; Dobash et al., 2009). The United Nations General Assembly (1993) defines it as an act of gender-based violence (GBV) that results in physical, sexual, mental harm, or suffering in both public and private life. This definition highlights that violence occurs due to inequalities between men and women. In many countries, the term DV is referred to as partner violence (PV) or intimate partner violence (IPV), violence in an intimate relationship by current or ex-partner (Ellsberg Mary, 2011). This definition also refers to violence against child or elderly people or violence by any member of a household (Wood & Sommers, 2011). According to World Health Organization (WHO, 2018), DV is violence against women (VAW) and is one of the most common forms of violence women experience worldwide. The typology of violence is divided into three categories based on who committed the violent act: self-directed violence, interpersonal violence, and collective violence. This definition of WHO encompasses the diverse realities of abuse within DV; hence it is conceptualized for this study.

Although violence can take place among same-sex, women to men, against older adults, and parent to children in the family (Gadd et al., 2002), women are uniquely vulnerable, with 35% (1 in 3) of women experiencing violence in their lifetime (WHO, 2018), and 38% of women murdered by their male partner worldwide (WHO, 2021). The most common forms of violence that women experience from current and former intimate partners are physical and sexual violence. Data based on the global prevalence of IPV, from 2000 to 2018 in 154 countries indicate that almost 1 in 3 women of age group 15 to 49 years, suffer from physical or sexual violence either from current or ex-partners at least once during their lifetime (WHO, 2021). Physical violence (31%) had a higher prevalence followed by sexual violence (18%) and stalking (10%). Violence committed against female (76%) is higher as compared to males (24%). In the global context, nearly 47,000 women and girls were killed by their partners or other family members in the year 2020, which is one girl killed every 11 minutes by her own family member (United Nations Office on Drugs and Crime [UNODC], 2021). Likewise, according to the Crime Survey for England and Wales, approximately 1.7 million (6.9%) women experienced DV in the year ending March 2022, as compared to 699,000 (3.0%) of men aged 16 years and older (Office for National Statistics [ONS], 2022). South Asian countries also have higher prevalence of DV (38%) (WHO, 2017) and Nepal is not an exception. It has a higher incidence of GBV and women are the main victims of male perpetrators (World Bank, 2018). According to a recent national survey, 26% of married women experienced physical, sexual, or emotional violence perpetrated by their husbands, 14% reported at least one-or-more forms of spousal violence in the past 12 months, and around 23% of all women experienced physical violence, 12% experienced emotional violence, and 7% experienced sexual violence (Ministry of Health [MOH], 2017, p. 343). This is a glimpse of gender inequality and discrimination against women (WHO, 2018). Not only does DV have a detrimental effect on women’s health, but it also has a huge financial and social impact on a country’s economy, with pressure on the health system, police force, criminal justice system, social services, and housing (Home Office, 2019). Furthermore, it has both long- and short-term effects on women, which influence her ability to take care of the kids and family and violet her basic human rights (Kennedy et al., 2020). Therefore, it is important to prevent or reduce the prevalence and severity of violence from happening and provide relevant service to those women who experience it (WHO, 2021). It is an ongoing major public health problem globally that needs to be addressed using a multisectoral approach to understand its features in-depth (WHO, 2021). This is only possible by identifying the context-specific risk factors for one’s exposure to DV/IPV to inform the prevention and intervention efforts, which highlight the significance of this review.

There are multifactorial reasons for DV in the context of Nepal. Since it is a Hindu country with patriarchal society, men are viewed as the head of the family and justified as a normal practice for them to perform violence to control and dominate women (York, 2011). Hence, inequality between gender is socially constructed and reinforced by cultural norms. Excessive alcohol consumption by husbands is one of the many reasons for DV/IPV in the context of Nepal and elsewhere, as it fluctuates the mood of the drinker and increases pre-existing anger and frustration (Atteraya et al., 2015; Bhatta et al., 2021; Clark et al., 2019; Deuba et al., 2016, Dhungel et al., 2017; Gautam & Jeong, 2019). Some studies have identified that educational deprivation (Lamichhane et al., 2021; Sapkota et al., 2016), and a lack of decision-making autonomy among women increase IPV victimization among Nepalese women (Atteraya et al., 2015). Early marriage (Pandey, 2016), earning less than husband, or depending on husband financially (Clark et al., 2019) are some other risk factors of DV/IPV. Witnessing violence during childhood or exposure to violence as a child are the associated risk factors for VAW both in Nepal and in a global context (Clark et al., 2019; Dalal et al., 2014; Pandey, 2016), including low- and lower middle-income countries (LALMIC) (Le et al., 2018). Nepal is one of the least developed countries in southeast Asia with low income (WHO, 2018). Children and young people who are maltreated or victimized are at increased risk of further victimization (Radford et al., 2013) or poly victimization (Hamby et al., 2012). Such children experience multiple forms of violence along their life course across multiple relationships with siblings, peers, dating partners, adults, and other family members (Hamby et al., 2012, p. 119). Study shows that 10% of children and adolescents aged 2 to 17 years experienced polyvictimization in the United States in the year 2006 (Finkelhor et al., 2005) and 10% of Spanish children aged 14 to 18 years (Soler et al., 2012). The prevalence is even higher (38%) in the LALMIC (Le et al., 2018). Polyvictimization is associated with higher risk of mental health and suicidal behaviors. Hence the time of exposure conceptualized for this study includes both the lifetime exposure and the exposure in the past year or last 12 months.

Nepal is divided into three geographical reasons: Mountains, Hills, and Terai. People living in the Terai region are associated with lower and untouchable caste also known as Terai caste or Dalits and are less privileged than other higher caste people such as Brahmin and Chettri that live in the Hills (Atteraya et al., 2015). Women belonging to the Terai region and of untouchable caste have higher prevalence of IPV than the Hindu women from high caste system living in the Hills. A cross-sectional study conducted among 905 participants which consists of urban poor (225) and general population (680) showed that prevalence of IPV was higher in urban poor (33.8%) than in general population (19.9%) (Oshiro et al., 2011). It is also common for Nepalese women to experience DV during pregnancy and the postpartum period (Bhatta & Assanangkornchai, 2019). A multicountry study conducted by WHO (2005) found that the prevalence of IPV was higher during pregnancy, ranging from as low as 1% in Japan to as high as 28% in Peru. A meta-analysis of 92 independent studies on DV during pregnancy shows higher prevalence (27.7%) in developing countries as compared to the developed countries (13.3%) (James et al., 2013). In a similar study from Nepal, the prevalence of DV was 27% among the pregnant women (Shrestha et al., 2016). In one of the studies conducted among 2,400 pregnant women of Nepal (Pun et al., 2019), there was an association of IPV during pregnancy resulting in adverse perinatal and maternal outcomes. Twenty percent of women reported experiencing violence during the pregnancy period thus giving birth to preterm infants. The reason for violence taking place during pregnancy is due to the fact that either the violence is initiated during pregnancy or it is the continuation of a previous episode of violence (Salazar et al., 2012). The main preparator in Nepalese households may not always be the partner but rather any member of the family (World Bank, 2018). In a study of 660 pregnant women visiting Paropakar Maternity and Women’s Hospital (PMWH), the prevalence of DV was higher (85.7%) among women who had controlling mothers-in-law (Bhatta & Assanangkornchai, 2019). Findings from the study on 357 men and women living in migrant communities of Baglung district, Nepal, shows that factors such as borrowing money or food, job seeking, unemployment stress, difficulties in accessing money, and mother in-law’s cruelty were associated with women’s increased risk of IPV (Shai et al., 2019). Young women who are of low socioeconomic status are more vulnerable to IPV as compared to the women who have their income and autonomy (Rishal et al., 2018). The reason for this could be that women with good social status contribute equally in terms of finance and decision-making, thus lowering their risk of IPV victimization.

### Current Review

Since the Nepal government passed the Domestic Violence Act in 2008, cases of DV are now discussed on a daily basis in local newspapers, courts, and public domains, highlighting a growing and serious public and legal issue (Pun et al., 2019; Sanjel, 2013). There is significant growth of primary studies on DV against women in Nepal. For example, several population-based studies have focused on the magnitude and factors associated with VAW in the context of Nepal (Gautam and Jeong, 2019; Lamichhane, Puri et al., 2011; Sapkota et al., 2016). However, there are no published systematic reviews on DV against women and associated factors in the context of Nepal. Thus, this study aims to fill this gap by bringing together and summarizing the context-specific determinants of DV against women in Nepal to inform the prevention and intervention efforts. Information from this review could be utilized to provide culturally nuanced services to support the needs of Nepalese women DV/IPV survivors which highlights the significance of this review.

### Social Ecological Framework

The factors that increase the risk of violence perpetration and victimization cannot be explained by a single factor alone and range from individual, family, community to wider society levels (WHO, 2012, p. 3). Therefore, this study utilizes ecological framework by Krug et al., (2002) to look at the risk factors as it considers multiple levels of influence and helps to explore how various factors interact and contribute to DV risk. Initially, this framework was introduced by Bronfenbrenner and Morris (1998) to study the risk factors contributing to IPV among women and girls. However, it is now used for similar studies in which women and children are exposed (Wilson, 2021). This framework views violence as the outcome of interaction of multiple factors at an individual, relationship, community, and societal level (Krug et al., 2002). At the individual level, this framework focuses on personal history and biological factors which influences the behavior of an individual and the likelihood of being a victim or perpetrating violence, (e.g., a child who witnesses violence in his earlier life grows up to become a perpetrator). At the relationship level, it looks at the relationships among family, friends, and intimate partners, and their influence on whether someone becomes a victim or perpetrator of violence. At the community level, it focuses on relationships with schools, neighborhoods, and workplaces. At a societal level, it focuses on whether violence is promoted from social factors such as inequalities, social-cultural norms such as males dominating women, cultural norms such as hiding the violence within a household and accepting it as normal. A study shows that women experiencing IPV continue to do so due to the interaction of multiple factors operating at multiple levels of the ecological framework than solely an individual factor (Sabri et al., 2018). Therefore, using this framework we hope to explore the risk factors for violence in multiple contexts of Nepalese women’s lives and identify their needs for services.

## Method

### Database Search

Violence was declared a major growing public health problem in 1996 and “The first world report on violence and health” was published by WHO in the year 2002 (Kurg et al., 2002) which is a comprehensive review of violence as a problem in the global context. Therefore, based on this timeline as a guide, key published literature in the Nepalese context from January 2000 to January 2022 has been identified and reviewed. Furthermore, studies on DV/IPV in Nepalese contest have evolved over time and gained recognition only recently. To provide insight into the current issues related to the topic, recent literature review is more likely to be relevant to the current context. Therefore, this timeframe was considered as it captures the recent developments and trends related to the topic. Studies published in English and conducted with Nepalese women were systematically searched using three search strategies. Firstly, databases PubMed, MEDLINE, CINAHL, and PsycINFO were searched. Secondly, Cochrane database was searched for gray literatures. Lastly, this was supplemented by manual searches of the reference lists in Google and Google Scholar. Any studies with female participants aged 15 to 49 years who experienced any form of violence (physical, sexual, and emotional/psychological) perpetrated by their husband, partner, ex-partner, in-laws, or any family members were included in this review. Findings from the Nepal Demographic Health Survey (MOH, 2017) were also included in the review.

In the context of Nepal, DV is known as “Gharelu hinsha” or “Mahila hinsha” which also translates to GBV (Clark et al., 2019, p. 1). IPV is relatively a new concept and considered a subset of GBV and is less understood and explored in Nepal (Ghimire and Samuels, 2017). Therefore, due to the indefinite terminologies surrounding the definition of DV, to study the risk factors of DV in depth, one cannot exclude studies on GBV, sexual violence, physical violence, VAW, and IPV. Hence this review utilizes studies on heterosexual women along with these terminologies and the same is adopted for the database search terms too. Hence the keywords used were:

- DV or physical violence or women abuse or PV or IPV or sexual violence or battered women and
- risk factor* or contributing factor* or predisposing factor* or predictor* or cause* or vulnerability factor* and
- Nepal*

Asterisks (*) were used for terms “factor” and “Nepal” to capture the variations of words or root and to capture all the possible forms of a term. For example, using asterisk in case of Nepal allows to capture the variations such as Nepali and Nepalese. The subject librarian was consulted in identifying these keywords.

To ensure we captured for IPV/DV articles that were not published in scholarly journals, we conducted manual search in google which resulted in one additional article for the review. We also searched gray literatures in Cochrane database which resulted in four articles that were already identified by previous databases search; hence no new articles were added.

### Eligibility Criteria

PICO framework was modified and adopted to assess the eligibility of the retrieved studies. It helps to break down the questions into small sections based on the components of population, intervention, comparison, and outcome. It gives clarity to the question and makes it specific to address the research problem. Since this was not an intervention study, PICO was adopted and used as such. The population (P) of interest were Nepalese women/girls aged 15 to 49 years. Estimate of the potential risk factors for DV such as individual factors, socioeconomic factors, and cultural norms were considered as intervention (I) or exposure. Since this was a review study, no comparison (C) was made or established. The outcome (O) considered was any form of violence or abuse such as physical violence, sexual violence, or emotional violence by husband and any other family members in the past year or lifetime. Since the purpose of this study was to bring together the risk factors of IPV/DV in Nepalese context, the timeline of exposure considered were both lifetime exposure and exposure in the past 12 months. The present review sought any studies on risk factors for intimate partner, domestic, and/or family violence in Nepal. Hence, studies on women with illnesses such as HIV, women in refuge, care homes or brothel, case report of a single women, and non-English studies were excluded, as they were not within the scope of this study and were taken as requiring a separate review.

### Study Selection

**Figure 1.**
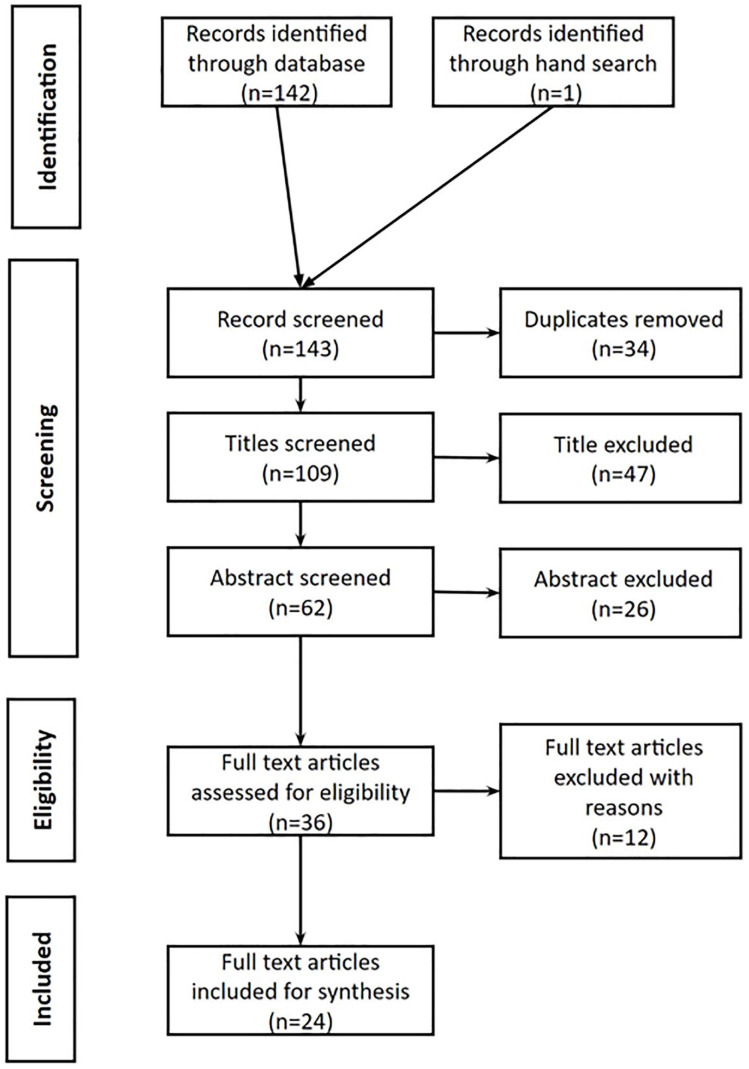
Process of study selection using the preferred reporting items for systematic reviews and meta-analyses statement.

The Preferred Reporting Items for Systematic Reviews and Meta-analyses (PRISMA) was used to shortlist studies based on the eligibility criteria (Liberati et al., 2009). A total of 143 studies were retrieved from the databases of which 34 were duplicates and 73 were ineligible based on their titles and abstracts. The titles that were irrelevant to the study were flagged up and excluded from the study after reading their abstracts. Of the 36 studies that qualified for a full text review, 12 were removed as they did not meet the eligibility criteria. This meant that a final of 24 studies were included for a full text review (see Figure 1). The first (BD) and second author (PS) reviewed the articles separately and checked whether they met the eligibility criteria, the decision was color coded in a Microsoft table, green for included, red for excluded, and amber for confusing studies. We (BD and PS) made a note next to each article with reasons for inclusion, exclusion, and confusion and discussed each reason. The discrepancies were resolved through discussion and consensus.

### Quality Assessment

The Mixed Methods Appraisal Tool (MMAT) (Hong et al., 2018) designed to assess the quality of quantitative, qualitative, and mixed method studies (Pace et al., 2012) was used to assess the quality of the 24 included studies. This helped to examine the studies systematically and ensured trustworthiness, validity, and reliability (Harden & Gough, 2012). Each study was rated as “yes,” “no,” or “can’t tell” based on the five core criteria of the MMAT guidelines (Hong et al., 2018). A score of 1 was assigned to each criterion that was met, and 0 was assigned if the criteria is not met or if it was unclear to the reviewer. Lower scores indicated poor quality and higher stars indicated a higher quality study. The total number of criteria met was tallied and the final quality rating of “low,” “moderate,” or “high” quality was given to each study according to the overall score. Since this was the first review study to assess the risk factors for violence in context of Nepal, we agreed to prioritize any studies with the outcome of DV/IPV. Hence, only the studies that scored less than 2 points were excluded. However, all the studies scored at least 2 scores in the MMAT criteria and eventually none were removed. Thus, all 24 studies were included in the final analysis. Of the 24 studies included, 2 studies were rated 2, 8 studies were rated 3, 13 studies were rated 4 scores and only 1 study was rated 5 scores. The first author (BD), with the help of the second author (PS), appraised the articles, and where it was difficult to decide, the discordance was adjudicated and verified after discussion.

### Data Extraction

After screening the articles, duplicate studies were removed by comparing authors’ names, the publication year, and title of the studies. The titles and abstract were critically reviewed using the PICO format. The articles were read and reread, and data were extracted in a Microsoft table based on the columns of authors’ name, year of publication, title, place of study, study objectives, design of study, sample size, and main findings. The data extracted from the selected studies were the characteristics of the study, prevalence of violence, and the associated factors. Reports of the magnitude of different types of violence and associated factors were extracted (see Table 1).

**Table 1.** Data Extraction Table With a Summary of the Study Characteristics and Main Findings.

Author/Title	Location	Sample	Major Finding (Factors Associated with Domestic Violence): 1- Individual, 2- Relationship, 3- Community, 4- Societal Level.
Adhikari and Tamang (2010)	Four districts of Nepal (Achham, Gulmi, Rupendehi, and Ilam)	-Cross-sectional survey with married women of reproductive age (15–49 years) - Random selection of 1,536 married women interviewed 48 clusters (12 clusters times 4 districts of Nepal)	(1) Literate status of women, decision- making capacity about their own health care, and higher level of alcohol consumption by husband, (2) having husbands who were 5 or more years older and (4) patriarchal control.
Atteraya et al. (2015)	Three geographical regions (Mountains, Hills, and Terai) and five development regions (East, West, Central, Mid-West, and Far-West region)	Random selection of 3,373 married women (mean age of women—32 years) - Nepal Demographic and Health Survey (NDHS) 2011, modified version of Conflict tactics scale (Straus, 1990) was utilized by NDHS - Cross-sectional	(1) Female illiteracy, low economic status, early marriage, age at marriage, violent family history, and lack of decision-making autonomy, (2) husband’s level of education, background (whether he was alcoholic), and higher no. of children. (3) women living in Terai region, belonging to underprivileged or untouchable caste and ethnic groups.
Bhatta and Assanangkornchai (2019)	Paropakar Maternity and Women’s Hospital (PMWH), Kathmandu, Nepal	-A total of 165 women aged 15–49 years who were in the trimester of pregnancy and postpartum attending the antenatal care and immunization clinic in PMWH -Purposive sample -Cross-sectional study using questionnaire adapted from WHO domestic violence against women instrument.	(1) Been married for 2 to 5 years, illiterate husbands, women in their first trimester of pregnancy, and previous history of domestic violence (DV), (2) controlling mother-in-law and belonging to the Janjati ethnic group.
Bhatta et al. (2021)	Antenatal care and postnatal care clinics of a government hospital in Kathmandu district, Nepal	660 women (aged 15–49) in pregnancy and postpartum periods. - Cross-sectional study Purposive	(1) Women whose husbands drank alcohol, time during second trimester of pregnancy, illiteracy of the women, duration of marriage 2–5 years, (2) husband with controlling manner and (3) belonging to Janjati ethnicity.
Clark et al. (2019)	Chitwan, Kapilvastu, and Nawalparasi district in Nepal	1,800 women 19 to 49 years old, from Nawalparasi, Chitwan, and Kapilvastu districts in Nepal - Cross-sectional - Simple random sample Community setting	(1) employment (less earnings than husband), financial stress, women whose husbands drunk frequently, exposure to intimate partner violence (IPV) as a child for both wife and husband, in-laws perpetrating violence and (3) caste (Disadvantage, Dalit, and Janjati).
Clark et al. (2018)	72 wards in three districts (Chitwan, Kapilvastu, and Nawalparasi) in Nepal	1,435 females, married, and of the reproductive age - Cross-sectional Random sampling	(1) husbands’ education level, perceived financial stress and (4) collective normative expectations.
Dalal et al. (2014)	75 districts of Nepal, 289 wards and sub wards in rural and urban area of Nepal.	Cross-sectional population-based study on 4,210 women of reproductive age (15–49 years) using data from the Nepal demographic and health survey 2011 (NDHS, 2017) Two stages of data collection: first stage- Probability proportional to size strategy and in second stage—random selection	(1) Women’s age (>40), level of education—no education or primary education, husbands’ level of education—no education and primary education, having alcoholic husband, family history of intimate partner violence against women (IPVAW), (2) decision-making capacity of women on contraception use, visiting friends or relatives, refusing sex and condom use, women with lower economic status, having a controlling husband, women afraid of their husband and (3) living in more disadvantaged neighborhood.
Deuba et al. (2016)	Urban slums in Kathmandu, Nepal	Qualitative study of 20 young pregnant women from 13 urban slums in the Kathmandu valley Purposive sampling	(1) husband with alcohol use disorder, (2) refusing to have sex, and economic dependence on husband. (4) identification of fetal gender and giving birth to a girl.
Dhungel et al. (2017)	Kathmandu, Nepal.	Cross-sectional study of 236 women aged 15 to 49 years old, working in carpet and garment factories. Convenience sampling	(1) Age of the woman (>29 years), alcohol consumption of the husband, education of husband above primary level, and (2) economic dependency on husband.
Diamond-Smith et al. (2019)	Three geographical regions (Mountains, Hills, Terai) and five development regions (East, West, Central, Mid-West, and Far-West region) of Nepal.	Data from the 2011 Nepal Demographic and Health Survey on 3,373 women of reproductive age (15–49 years) - Cross-sectional Stratified, two-stage cluster design	(1) Food insecurity and (2) women’s level of household decision-making capacity.
Gautam and Jeong (2019)	Urban and rural areas Nepal	Cross-sectional study conducted from the secondary data of Nepal Demographic and Health Survey (NDHS) 2016. A total of 11,040 households were selected of which 12,862 were women and 4,063 were men aged 15 to 49 years old. -Multistage cluster sampling	(1) women whose husbands get drunk very often, (2) women whose husbands show marital control behaviors and are afraid of their husbands.
Lamichhane et al. (2021)	75 districts of Nepal	Interactive Voice Response survey among 1,314 adolescent girls and young women aged 18–24 years. - Cross-sectional study - Descriptive analysis was used. Random sampling	(1) girls with no formal education, age range of 22–24 years, and (3) belonging to Dalit (low caste) group.
Lamichhane, Puri et al. (2011)	Four districts—Dolkha, Sindhupalchow, Dang, and Kapilvastu of Nepal	Cross-sectional study was conducted in 2009 among 1,296 young married women aged 15 to 24 years in four major ethnic groups. Two-stage random sampling	(1) illiteracy, women in agriculture/daily wages/poultry farming, (2) No or little interspousal communication and lower autonomy of women.
Ministry of Health (2017)	Country-wide survey.	11,040 households were successfully interviewed of which 12,862 were women. - Cross-sectional Random sampling	(1) women’s level of education, husband’s higher level of alcohol consumption, women who witness their fathers beat mothers (2) controlling husband, and (4) norms accepting that wife beating was justified in specified situations such as if she burns the food, argues with him, goes out without telling him, neglects the children, or refuses to have sex with him.
Oshiro et al. (2011)	Kathmandu, Nepal.	A cross-sectional study was conducted by structured questionnaire interview. Participants included 905 ever-married women in Kathmandu aged 15 to 49 years. - Random selection Community based	(1) frequency of husband’s drinking, husband’s lower level of educational, lower household economic status and early marriage (3) living in urban poor area and (4) polygamy.
Pandey (2016)	10,826 households throughout Nepal.	Nationally representative sample of 3,373 women from Nepal Demographic and Health Survey, 2011. - Cross-sectional - Two-stage, stratified sample design Random sampling	(1) women’s age at the time of marriage (women who married at the age of 20 were more likely to experience physical and sexual abuse than those married at the age of 15), excessive consumption of alcohol, women witnessing their fathers abuse their mothers physically, (2) husband humiliating wives in front of others and (3) living in Terai region.
Puri et al. (2015)	Three districts of Nepal, Bhaktapur, Kaski, and Jhapa.	Cross-sectional survey of 475 women with a disability aged 16 years and above - Mutistage sampling Purposive sampling	(1) age: women aged <20 were 6.9 times more likely to experience lifetime violence compared with women aged 65 and above, disability and (2) controlling husband.
Puri et al. (2016)	Two districts (Dang and Tanahu) of Nepal.	75 eligible respondents of which 39 were women and 36 were men. The participants were identified via home visiting. The Causal Flow Analysis (CFA) was conducted. - Qualitative - Participatory approach The CFA	(1) lack of education of husband, alcohol consumption, (2) women’s inability to negotiate, no income, economic dependency of women, husband being inconsiderate, (4) extra marital affair of wife or husband, masculine ideals among men, fear of having co-wife and patriarchal society.
Sapkota et al. (2016)	Kuleshwor and Sindhuli	Community-based cross-sectional study was conducted among 355 women of reproductive age. Random sampling	(1) alcohol use, women having poor mental health, women with no formal education and unemployment status, (2) women with controlling husband, (4) norms supporting violence and accepting violence as normal.
Shai et al. (2019)	Bhimapokhara and Resha, the two migrant communities in the Baglung district of Nepal.	Cross-sectional quantitative study on 357 participants of which 100 were daughters-in-law, 100 mothers-in-law, 79 husbands, and 78 fathers-in-law. Multistage sampling with stratification by district (random)	(1) old age of women, husband experiencing childhood trauma and depression (2) having controlling husband, poor relation with husband and mother-in-law.
Shrestha et al. (2016)	TUTH, a tertiary level health care facilities in Kathmandu, Nepal.	A descriptive cross-sectional study was conducted among 404 pregnant women in their third trimester of pregnancy. - Convenient sampling was used to select the study population. Healthcare setting	(1) husbands age (25–34 years), women married for 2 to 5 years and who had one or two children, experience of violence before the current pregnancy and (2) husband’s controlling behavior.
Silwal and Thapa (2020)	Paropakar Maternity and Women’s Hospital (PMWH), Tertiary hospital, Nepal	A descriptive cross-sectional study was conducted on 112 women attending the subfertility clinic. Face-to-face interview was conducted using a structured interview schedule. - Convenient sampling was used. Hospital setting.	(1) infertility—the period of secondary infertility than the primary infertility.
Yoshikawa et al. (2014)	Kirtipur municipality and Bhaktapur district of Nepal.	Cross-sectional study on 717 couples with wives aged 18 to 49 years. - Randomly selected. Community based.	(1) husband witnessing violence on mother, husband experiencing violence during childhood and (4) husband’s acceptance of wife beating.
Yoshikawa et al. (2018)	Kritipur municipality and Bhaktapur district in Kathmandu.	Community-based cross-sectional study with 717 couples aged 18 to 49 years. Random selection	(1) husbands’ age and education, household income (3) place of residency and wives’ caste.

## Results

### Characteristics of Studies

The result section of this article is based on the findings from 24 studies included in the review. The evidence derived from these studies are heterogenous and are based on the research objectives, methods, and outcome measured. Of the 24 studies included in the analysis, two were qualitative (Deuba et al., 2016; Puri et al., 2016) and 22 were cross-sectional studies. In most of the studies, the participants were women aged 16 to 49 years. Since most of the studies were cross-sectional, the samples were random and from the general population. Four of the studies had purposive sample (Bhatta & Assanangkornchai, 2019; Bhatta et al., 2021; Deuba et al., 2016; Puri et al., 2015). Three studies had convenient (Dhungel et al., 2017; Shrestha et al., 2016; Silwal & Thapa, 2020) and one had participatory samples (Puri et al., 2016). Four of the studies had samples from healthcare settings (Bhatta & Assanangkornchai, 2019; Bhatta et al., 2021; Shrestha et al., 2016; Silwal & Thapa, 2020) and one had sample form the slums (Deuba et al., 2016). Rest of the study had their samples from the community or general population. Four studies (Bhatta & Assanangkornchai, 2019, Bhatta et al., 2021, Deuba et al., 2016; Shrestha et al., 2016) examined women’s experience of IPV during pregnancy. Most of the studies included religion and where religion was not included either caste or ethnicity was included. Only 3 of 24 studies did not include either of the caste, religion, or ethnicity (Adhikari & Tamang, 2010; Clark et al., 2018; Dhungel et al., 2017). The study has highlighted that the multiple forms of violence that women experience throughout their lives and the perpetrators also vary. Different forms of violence that women experienced were physical, sexual, and emotional IPV, any form of IPV, different types of IPV, different violent act, any form of violence and physical or sexual abuse. Most of the violent acts were committed by their partners followed by in-laws, family, friends, and neighbors. Women with disability were more likely to experience violence from not just the family members (58%), neighbors (52.6%), and intimate partners (39.1%) but also from strangers (12.8%) and friends (8%) (Puri et al., 2015).

The tools used were individual and group interviews, survey questionnaires, and short form of Conflict tactics scale (CTS2). Most of the studies mention obtaining ethical approval except two (Dhungel et al., 2017; Pandey, 2016). However, the study participants were informed about the study and that their participation was voluntary. All the studies had a clear outcome set prior to the study. Sample sizes vary from as low as 20 (Deuba et al., 2016), to as many as 12,862 (Gautam & Jeong, 2019) women of reproductive age group. Where the participants are recruited from general population settings, the sample size was higher compared to the participants recruited from the specific settings such as slums and healthcare settings.

The analysis found that factors operating at multiple contexts of ecological framework and their interactions contributed as a risk factor of violence against Nepalese women (Table 2). Number of studies shows that approximately half of the women participants in the studies experienced different forms of violence in their lifetimes (Adhikari & Tamang, 2010; Lamichhane, Puri et al., 2011; Puri et al., 2015; Silwal & Thapa, 2020). The most prevalent violence was physical violence followed by sexual and emotional violence. The perpetrators were husbands, family members, in-laws, friends, and neighbors. Individual-level risk factors and relationship-level risk factors were reported in most of the studies followed by the community- and societal-level risk factors. In total 38 risk factors were reported.

**Table 2.** Identified Risk Factors Categorized Using the Ecological Model.

Risk Factors Associated With Domestic Violence. (1- Individual, 2- Relationship, 3- Community, 4- Societal Level)	Citations	No. of Studies
1. Individual level		
Literacy level of women	Adhikari and Tamang (2010), Lamichhane et al. (2011), Dalal et al. (2014), Atteraya et al. (2015), Sapkota et al. (2016), MOH (2017), Bhatta et al. (2021), Lamichhane et al. (2021).	8
Pregnancy and postpartum period	Shrestha et al. (2016), Bhatta and Assanangkornchai (2019), Bhatta et al. (2021).	3
Women’s age	Dalal et al. (2014), Puri et al. (2015), Dhungel et al. (2017), Shai et al. (2019).	4
Women with poor mental health	Sapkota et al. (2016).	1
Alcohol consumption level of husband	Adhikari and Tamang (2010), Oshiro et al. (2011), Dalal et al. (2014), Atteraya et al. (2015), Deuba et al. (2016), Pandey (2016), Puri et al. (2016), Sapkota et al. (2016), Dhungel et al. (2017), MOH (2017), Clark et al. (2019), Gautam and Jeong (2019), Bhatta et al. (2021).	13
Husband’s level of education	Oshiro et al. (2011), Dalal et al. (2014), Atteraya et al. (2015), Puri et al. (2016), Dhungel et al. (2017), Clark et al. (2018), Yoshikawa et al. (2018), Bhatta and Assanangkornchai (2019).	8
Husband witnessing violence on mother/childhood experience	Yoshikawa et al. (2014).	1
Early marriage/age at marriage	Oshiro et al. (2011), Atteraya et al. (2015), Pandey (2016).	3
Duration of marriage	Bhatta and Assanangkornchai (2019), Bhatta et al. (2021).	2
Economic status (lower)	Oshiro et al. (2011), Dalal et al. (2014), Atteraya et al. (2015).	3
Violent family history/witnessing violence at childhood/exposure to intimate partner violence as a child	Dalal et al. (2014), Atteraya et al. (2015), Pandey (2016), MOH (2017), Clark et al. (2019), Shai et al. (2019).	6
Women in agriculture/daily wages/poultry farming	Lamichhane et al. (2011).	1
Employment status/unemployed Earning less than husband	Sapkota et al. (2016), Clark et al. (2019).	2
Food insecurity	Deuba et al. (2016), Diamond-Smith et al. (2019), Shai et al. (2019).	3
Disability	Puri et al. (2015).	1
Infertility	Silwal and Thapa (2020).	1
2. Relationship level		
Decision-making capacity	Adhikari and Tamang (2010), Dalal et al. (2014), Atteraya et al. (2015), Puri et al. (2015), Diamond-Smith et al. (2019).	5
Lower autonomy	Lamichhane et al. (2011).	1
Women afraid of their husbands	Dalal et al. (2014), Gautam and Jeong (2019).	2
Husband and wife age difference (>5 years or older)	Adhikari and Tamang (2010).	1
Higher number of children	Atteraya et al. (2015).	1
Experiencing violence from in-laws	Clark et al. (2019).	1
Controlling husband	Dalal et al. (2014), Puri et al. (2015), Sapkota et al. (2016), Puri et al. (2016), Shrestha et al. (2016), MOH (2017), Gautam and Jeong (2019), Shai et al. (2019), Bhatta et al. (2021).	9
Extra marital affair of wife or husband	Puri et al. (2016).	1
Fear of having co-wife	Puri et al. (2016).	1
Controlling mother-in-law	Bhatta and Assanangkornchai (2019).	1
Financial stress	Clark et al. (2018), Yoshikawa et al. (2018), Clark et al. (2019).	3
Economic dependency on husband/no income of women	Deuba et al. (2016), Puri et al. (2016), Dhungel et al. (2017).	3
Refusing to have sex	Deuba et al. (2016).	1
No or little interspousal communication	Lamichhane et al. (2011).	1
Poor relationship with husband and mother-in-law	Shai et al. (2019).	1
3. Community level		
Living in Terai region	Atteraya et al. (2015), Pandey (2016).	2
Living in disadvantaged neighborhood	Dalal et al. (2014).	1
Caste belonging to underprivileged or untouchable caste and ethnic group	Atteraya et al. (2015), Bhatta and Assanangkornchai (2019), Clark et al. (2019), Bhatta et al. (2021), Lamichhane et al. (2021).	5
4. Societal level		
Patriarchal control	Adhikari and Tamang (2010).	1
Patriarchal society	Puri et al. (2016).	1
Polygamy	Oshiro et al. (2011).	1
Giving birth to a girl/identification of fetal gender	Deuba et al. (2016).	1

### Individual Level

Individual-level risk factors include both victims’ and perpetrators’ demographics and individual factors that increase the risk of victimization or perpetration. Alcohol consumption by husbands was identified in 13 of 24 studies as the most prevalent risk factor of violence (Adhikari & Tamang, 2010; Atteraya et al., 2015; Bhatta et al., 2021; Clark et al., 2019; Dalal et al., 2014; Deuba et al., 2016; Dhungel et al., 2017; Gautam & Jeong, 2019; MOH, 2017; Oshiro et al., 2011; Pandey, 2016; Puri et al., 2016; Sapkota et al., 2016).

Both the husbands’ and wives’ education levels were another major factor reported in the studies, with husbands’ illiteracy or lower level of education identified as a risk factor in 8 of 24 studies (Atteraya et al., 2015; Bhatta & Assanangkornchai, 2019; Clark et. al., 2018; Dalal et al., 2014; Dhungel et al., 2017; Oshiro et al., 2011; Puri et al., 2016; Yoshikawa et al., 2018). Women married to illiterate husbands were twice as likely to experience DV (AOR = 1.78; 95% CI [1.05, 3.01]) than women married to literate husbands (Bhatta & Assanangkornchai, 2019). Similarly, women’s literacy levels were also an associated factor in 8 of 24 studies (Adhikari & Tamang, 2010; Atteraya et al., 2015; Bhatta et al., 2021; Dalal et al., 2014; Lamichhane, Puri et al., 2011; Lamichhane et al., 2021; MOH, 2017; Sapkota et al., 2016). However, both husbands’ and wives’ levels of education above the primary level were a protective factor in some studies (Dalal et al., 2014; Dhungel et al., 2017).

The time of pregnancy and postpartum were also contributing factors at the individual level for VAW (Bhatta & Assanangkornchai, 2019; Bhatta et al., 2021; Shrestha et al., 2016), with the most affected period was second trimester of pregnancy in two of the studies (Bhatta & Assanangkornchai, 2019; Bhatta et al., 2021). An 18-year-old women who was 8 months pregnant describes her story as:

I have been being beaten for 3 years (during pregnancies). My husband wants me to give birth to sons; but I am unlucky. I am not able to give him a son, I have only given birth to daughters during the 3 years. I have 3 daughters already and this one is also a daughter (pointing out abdomen). We went for sex identification one month ago and came to know that this one is also a daughter. Since that day, he (husband) started drinking in unlimited amounts. He comes home at midnight and beats me with such a big stick (showing the size with her hand). I don’t even remember the number of times he beat me because he beats me till he cools down and blame my maternal home for giving birth to me. I feel so sad, and I regret marrying him (tears in her eyes). . .. (Deuba et al., 2016, p. 5)

This statement illustrates that violence in the context of Nepal is multifaceted and is not the outcome of an individual factor alone. A number of risk factors such as the husband’s level of alcohol consumption (individual level), the illiteracy of women (individual level), and patriarchy (societal level), are collective risk factors in the above statement.

Both husbands’ and wives’ exposures to violence or witnessing violence during childhood were another important factor for perpetrating and experiencing violence (Atteraya et al., 2015; Clark et al., 2019; Dalal et al., 2014; MOH, 2017; Pandey, 2016; Shai et al., 2019). Age of a women was also a key factor for VAW (Dalal et al., 2014; Dhungel et al., 2017; Puri et al., 2015; Shai et al., 2019). Women’s higher age (>29) was a risk factor for physical violence in one study (Dhungel et al., 2017) and in another study, the prevalence of emotional, physical, and sexual violence was higher on women who were 40 years and older compared to their counterparts (Dalal et al., 2014). However, finding from other studies show that girls marrying at an early age were also particularly prone to physical and sexual violence (Pandey, 2016; Puri et al., 2016). Other identified risk factors at the individual level included women’s poor mental health (Sapkota et al., 2016), food insecurity (Diamond-Smith et al., 2019), disability (Puri et al., 2015), and infertility (Silwal & Thapa, 2020).

### Relationship Level

At the relationship level, lack of decision-making autonomy of women or level of autonomy, both high and low, toward the household, relationship, or own health were the most prevalent risk factors identified in five studies (Adhikari & Tamang, 2010; Atteraya et al., 2015; Dalal et al., 2014; Diamond-Smith et al., 2019; Puri et al., 2015). Women with lower autonomy were less able to communicate well with their husbands, which is a risk factor for violence (Lamichhane, Puri et al., 2011). Having a controlling husband (Bhatta et al., 2021; Dalal et al., 2014; Gautam & Jeong, 2019; MOH, 2017; Puri et al., 2015, 2016; Sapkota et al., 2016; Shai et al., 2019; Shrestha et al., 2016) and in-laws (Clark et al., 2019) increased the violence exposure among women. The perpetrators for women with disability were the family members, neighbors, intimate partners, and friends (Puri et al., 2016). Financial dependency on husbands (Deuba et al., 2016; Dhungel et al., 2017; Puri et al., 2016) and financial stress (Clark et al., 2018, 2019; Yoshikawa et al., 2018) have been found to increase the risk of IPV. Higher number of children (Atteraya et al., 2015), poor relationship with in-laws and husband (Shai et al., 2019) and having husbands who were older than wives (Adhikari & Tamang, 2010) were other contributing factors for violence.

### Community Level

At the community level, living in the Terai region (Atteraya et al., 2015; Pandey, 2016), disadvantaged neighborhoods (Dalal et al., 2014) or belonging to underprivileged or untouchable caste/ethnic groups (Atteraya et al., 2015; Bhatta & Assanangkornchai, 2019; Bhatta et al., 2021; Clark et al., 2019; Lamichhane et al., 2021) were identified as the risk factors. In the context of Nepalese culture, women belonging to Terai region and/or untouchable or underprivileged caste groups are more vulnerable to violence than the upper Hindu caste such as Brahmin and Chettri due to their lower socioeconomic status (Atteraya et al., 2015). Nepal is categorized into three geographical regions: Mountains, Hills, and Terai region. The prevalence of IPV is higher among women living in Terai region than in other two regions (Atteraya et al., 2015). Women and girls belonging to untouchable castes and lower ethnic groups faced discrimination due to their lower social status, lower literacy rates, and higher vulnerability to early marriage, which ultimately magnified their vulnerability to physical violence (Lamichanne et al., 2021).

### Societal Level

Risk factors at societal levels included patriarchal beliefs and norms that normalized violence (Adhikari & Tamang, 2010; Oshiro et al., 2011; Puri et al., 2016). Wife beating is considered a normal practice and perceived as justified by men in certain situations such as if women burn food, argue with husbands, leave their houses without telling men, perceived to have neglected children, or refused to engage in sexual intercourse (Clark et al., 2018; MOH, 2017; Pun et al., 2020; Yoshikawa et al. 2014). Higher level of patriarchal control by husbands (Adhikari & Tamang, 2010) and existence of traditional practices such as child/adolescent marriage, forced marriage, and polygamy were found to be the underlying factors of sexual violence among young women and girls (Puri et al., 2016). A 22-year-old participant who was married young at the age of 14 exemplified how she lacked the ability to negotiate relationship and sexuality due to her physical and mental immaturity as her husband forcefully performed sexual act on her and did not stop despite her screaming and crying and she bled (Puri et al., 2016).

## Discussion

A systematic review of the literature was conducted to examine the risk factor for VAW in the Nepalese context. Number of risk factors were identified at four different levels of ecological framework with majority of factors recorded at the individual and relationship level. The finding makes a strong contribution toward the exploration of risk factors in Nepalese culture-specific contexts. The result support existing literature on VAW which identify education, alcohol consumption level of husband, one’s childhood experience or witnessing of violence, period of pregnancy, food insecurity, norms supporting violence, and patriarchal control as risk factors beyond Nepalese context in multicultural settings. Additionally in the Nepalese context, women living in Terai region, belonging to underprivileged or lower caste or ethnic group, giving birth to daughters, tradition of childhood/adolescent or forced marriage, and practice of polygamy are some of the culture-specific risk factors for experiencing violence.

At the individual level, the alcohol consumption level of husband was identified as highly prevalent risk factor. Women whose husbands consumed alcohol were more likely to experience sexual coercion compared to their counterparts (Adhikari & Tamang, 2010). On the other hand, literacy, with the decision-making capacity of a woman, was identified as a protective factor against sexual coercion (Adhikari and Tamang, 2010). Women who could not refuse sex or felt unable to ask their partners to use contraception had a higher exposure to physical violence (Dalal et al., 2014). Both the husband and wives’ education levels were also a major contributing factor for VAW in number of studies. Having a lower level of education meant that women were either unemployed (Clark et al., 2019) or earned less than their husbands (Lamichhane, Puri et al., 2011; Sapkota et al., 2016), thus making minimum financial contributions to the family. Being unemployed not only involves a lack of social security but can also cause acute stress, which may lead to conflict and aggressive behaviors within a couple (Capaldi et al., 2012). However, it is important to note that women with higher education and employment are also at risk of violence from their partners (Cwikel et al., 2003). Some studies in the international context have found no association between women’s education and IPV (Bhattacharyya et al., 2011; Hindin & Adair, 2002; Panda & Agarwal, 2005) and suggest IPV may be the outcome of various factors in women’s lives, such as their childhood experiences and family background (Cueto et al., 2014; Dercon & Krishnan, 2009). Similarly, IPV in the context of Nepal is not the outcome of factors in women’s lives only. Husband-related factors were found to have a stronger significant association with women experiencing IPV than with women’s empowerment indicators (Gautam & Jeong, 2019). Therefore, interventions for reducing IPV need to emphasize involving both men and women.

In addition, a significant number of studies found that women experienced violence during pregnancy and postpartum period. Time during pregnancy and postpartum was a contributing factor for violence, attributed to the continuation of violence prior to pregnancy and women refusing to have sex during this period (Bhatta & Assanangkornchai, 2019). Similar findings were observed in an international study based on data from over 50 countries on pregnant women that showed that one-in-ten mothers were exposed to physical violence, one-in-five to psychological abuse, and one-in-twenty to sexual violence perpetrated by partners (Román-Gálvez et al., 2021). In the context of Nepal, other contributing factors during pregnancy and postpartum were the identification of the fetal gender and giving birth to a daughter (Deuba et al., 2016). This could be because Nepal is a Hindu country and in Hinduism, it is the son who performs the last ritual of the parents upon death. The religious belief that sons transcend the ancestors to the next world after death emphasizes the importance of having a son over daughter (Pun et al., 2020). Despite the laws in Nepal that prohibit the identification of fetal gender and sex-selective abortion, these practices continue to be prevalent. This can be attributed to the widespread availability of technology and a strong cultural preference for males (Lamichhane, Harken et al., 2011). Nepali society fosters high value for son as it is a Hindu society with patrilineal structure. The sons perform funeral rites, bring wife and dowery to the family, continue family name, and support parents with resources in old days. Stressing the value of male child increases the nonconsensual sex among couples (Puri et al., 2016). Even if the couple has a desirable number of children but the wife or husband still wants a son, and either of the partners does not want a son, then nonconsensual sex can take place, leading to sexual exploitation, that is, forcing someone for sex to have son. One of the examples of men stressing the value for son is illustrated here “Those who have buffaloes won the jungle and those who have sons have money” (Puri et al., 2016, p. 14). In a setting where no preference is given to the gender of a child and both male and female child are treated equally, this may not be applicable.

This review has also identified the association between IPV and food insecurity which mirrors the finding from the global context too (Bloom et al., 2020; Choudhary et al., 2020). IPV due to food insecurity is suggested to have intergenerational effects meaning children born from such parents are also likely to be impacted (Murray et al., 2018). Furthermore, it effects marginalized communities disproportionately (Nagata et al., 2019). Since Nepal has many marginalized communities, it is important to conduct more research on this issue as food insecurity is associated with excessive amount of negative physical and mental health outcomes (Dutton, 2009).

The existence of traditional practices such as child/adolescent marriage, forced marriage, and polygamy are the underlying factors for sexual violence among young women and girls in Nepal (Puri et al., 2016). Girls who are married at an early age are considered immature both physically and mentally and unable to negotiate relationships leaving them prone to sexual violence (Puri et al., 2016). Findings from the review also suggests that women marrying at an early age or below 20 years (Pandey, 2016) are at a higher risk of experiencing violence, particularly physical and sexual abuse, with the prevalence increasing after 2 to 5 years of marriage (Adhikari & Tamang, 2010). Women who marry early tend to have lower education levels and larger numbers of children, which are identified as risk factors for experiencing violence (Oshiro et al., 2011). However, this review also identified that older women were also equally prone to physical violence (Dhungel et al., 2017) implying that older age of women may not protect them from the risk of violence. Hence, to determine whether age of a women is a risk factor it may need to be coupled with other factors such as the age when she got married, number of children, whether she has a controlling husband, and her decision-making capacity. Additionally, women whose husbands are 5 or more years older are at a higher risk of sexual coercion (Adhikari & Tamang, 2010). Overall, studies show that early marriage is linked with a higher prevalence of physical violence (Atteraya et al., 2015; Oshiro et al., 2011; Pandey, 2016) and the average age of first abuse among once married women globally is 22.1 years, with abuse starting on an average 3.5 (2.5–5.2) years after marriage (Peterman et. al., 2015). It is possible that these young women and girls have negative effect in their emotional health. Most studies included in the present review looked at physical and sexual violence only. Therefore, it was not possible to assess risk factors for emotional abuse. Forthcoming studies need to consider assessing emotional abuse alongside physical and sexual abuse.

Both husbands’ and wives’ exposure to violence or witnessing violence during childhood was another important factor that triggered VAW. This is consistent with findings from other studies in different national contexts (Bell & Naugle, 2008; Capaldi et al., 2012; Widom et al., 2014) and can be contextualized by Nepal being a patriarchal society where men dominating women is accepted as a social norm (Puri et al., 2016). As noted, DV is considered justified in Nepalese culture in certain situations such as if women burn food, argue with spouses, are perceived as neglecting children, exhibit independence by leaving their homes without informing males, or refuse to engage in sexual intercourse (Clark et al., 2018; MOH, 2017; Pun et.al., 2020; Yoshikawa et al., 2014). This indicates that male dominance is deeply rooted in Nepalese culture and society (Atteraya et al., 2015). In a review of IPV studies from 67 countries, it was found that nearly 50% participants justified IPV in at least one scenario and the most justified scenario were if women neglect her children, and goes out without telling her husband (Waltermaurer, 2012). In Ethiopia, similar findings were observed, where one-third of women accepted DV as normal if the husband had a perceived justification, like the findings discussed above (Semahegn & Mengistie, 2015). Women who experienced patriarchal control were more likely to experience sexual coercion by her husband compared to their counterpart (Adhikari & Tamang, 2010). This patriarchal control is a risk factor at a societal level, which influences the risk factors at an individual level. This is a clear example of how the interplay of risk factors at various levels of ecological framework influences VAW. Overall, the influence of patriarchy and societal acceptance of male perpetuation of VAW are in line with the findings from studies among South Asian women more generally (Kallivayalil, 2010; Patel, 2013; Sabri et al., 2018). A study conducted in Nepal, on 717 randomly selected couples identified a discordance in the reporting of IPV between wives and their husbands (Yoshikawa et al., 2018). The husband reported more violence against wives than wives did against their perpetrating husbands. This supports the notion that DV is accepted as normal yet remains hidden in Nepalese society. Additionally, collection of data from both husband and wives at the same time could have resulted in underreporting as the wives may be afraid that their husband would be informed of their responses. Despite assurance of confidentiality and anonymity, the wives in the study may have felt insecure and reluctant to share their experiences. Furthermore, social desirability bias could have occurred (Yoshikawa et al., 2018). This could also be attributed to the fact that patriarchal societies often hold women responsible for the failed relationship and demand them to be submissive toward their husbands which influence perceptions that VAW is normal (Siddiqui, 2014). Hence, they are likely to accept violence as normal and not report it. However, this study contradicts with findings from other similar studies which showed that perpetrators tend to underreport their violence against their partners (Schluter et al., 2007; Simpson & Christensen, 2005).

At the relationship level, the decision-making capacity of women was a risk factor for violence. Women with higher decision-making powers in the household were more likely to report emotional, sexual, and physical IPV since making decisions was perceived as triggering spousal tensions. However, findings from Puri et al.’s (2015) study note that women who make joint decisions with their husbands were less likely to experience violence during their lifetime, suggesting that decision-making powers of women can be both a risk and protective factor for violence. Future studies need to assess whether decision-making capacity of a women plays a protective role in reducing VAW.

Other identified risk factors for violence at the relationship level were financial stress, and dependency on husband. Finding from a study shows that women who depend upon their husbands financially, tolerate and accept violence as a strategy of coping mechanism (Deuba et al., 2016). Women who were earning less than or equivalent to their husbands and experiencing financial stress were associated with higher odds of experiencing IPV (Clark et al., 2019).

Similarly, controlling husbands and mothers-in-law were found to be risk factors for VAW. A study conducted in clinical setting found that women with controlling husbands were five times more likely to suffer from DV (Bhatta et al., 2021). Martin et al. (2002), (as cited in Gautam & Jeong, 2019, p. 16), argued that husbands with lower level of education tend to believe that it is considered justified to control their wives using the physical force to achieve domination. In addition, studies on South Asian women show that abuse from in-laws can contribute to violence (Raj et al., 2006). None or minimal interspousal communication and lower autonomy of women significantly increased the odds of married women experiencing violence (Lamichanne et al., 2011). Globally, where in-laws exercise their power over the couple, violence takes place either due to direct violence or instigation by sons (Clark et al., 2009; Ghimire & Samuels, 2017). One study found that living in a joint household was a protective factor for emotional IPV but not sexual or physical IPV (Diamond-Smith et al., 2019). It could be that couples were less comfortable arguing about personal issues in front of relatives due to fear of gossip which could have led to less emotional IPV. Another study also found that having a kind mother-in-law was a protective factor for IPV (Shai et al., 2019). Similarly, women with patriarchal gender norms and attitude were a protective factor against IPV (Shai et al., 2019) as they tend to excuse male dominance and tolerate gender inequalities and VAW.

Risk factors prevalent at the community level were belonging to underprivileged or untouchable castes, living in the Terai region, and living in disadvantaged neighborhoods. More recently, women from Dalit or lower community, aged 22 to 24 years were more likely to experience sexual violence as compared to women of 18 to 21 years of age (Lamichhane et al., 2021) within a context of COVID-19 pandemic. This is attributed to disruption in health service provisions and school closures (Lamichhane et al., 2021; Poudel et al., 2020), as schools are considered a safe place for girls and women. Discontinuation of education during COVID-19 means women and girls have been housebound which is where violence ordinarily takes place. Dalit women and girls are socially marginalized group and face discrimination due to their low social status, lower literacy rate, and higher vulnerability to early marriage. These factors could therefore have been exacerbated by social restrictions during the pandemic, thus increasing their vulnerability to physical violence (Lamichane et al., 2021). While concerning, the easing of restrictions and reopening of schools and healthcare service might be minimizing the vulnerability of Dalit girls and women to violence.

Most of the risk factors in this review were identified at the individual and relationship levels, and very few on the community and societal levels. Only few studies (*n* = 6) assessed the emotional abuse as compared to physical and sexual abuse. This is consequently an area that requires consideration in future studies. In general, this review has highlighted risk factors at four levels of the ecological framework (individual, relationship, community and societal) that can be used to address the VAW in Nepal and to develop and implement interventions and policies. Some of these risk factors are consistent with those observed in other countries thus can be used to predict VAW. Some of the factors such as education level, decision-making capacity, mother in-law, living in the joint family, patriarchal gender norms and attitude were identified as both risk and protective factors for VAW in Nepal.

## Conclusion

This analysis ultimately shows that the risk factors of VAW are the interplay of multiple factors at various levels of the ecological framework, and that intervention at one level could influence and minimize outcomes at the other levels. Although violence occurs due to factors at all the four levels of ecological framework, this review has shown that the most common are at individual and relationship levels and that husband-related factors are strongly associated than that of women. Thus, policy-makers at national, supranational, and governance levels would benefit from targeting intervention and health promotion programs toward youth population (<18 years) both male and female by emphasizing socially accepted norms and values that promote mutual respect among couples and minimize the prevalence of VAW in context of Nepal.

### Critical Findings

- VAW is a significant issue among Nepalese women with variation in the prevalence, risk factors, and types of violence.
- VAW in context of Nepal, is driven by the interaction of multiple factors operating at the individual, relationship, community, and societal level.

### Strength and Limitation of the Study

A major strength of this study is that it provides important implications for future research by bringing together all the studies on VAW in the context of Nepal and categorizing the risk factors according to the ecological model. Since most of the studies used were cross-sectional survey designs that might induce social desirability bias during self-reporting of violence because of cultural norms around disclosing sensitive and family secrets. The women’s concern of their own safety and stigma may hinder their true revelation of IPV/DV experience. Moreover, the studies used in this review were published in English meaning potentially important studies published in Nepalese may have been missed. Some other limitations to be considered are that this study focused extensively on heterosexual women only and does not include studies on LGBTQ. A study shows that LGBTQ people are more likely to suffer from IPV/DV experience as compared to the heterosexual individuals (Messinger, 2017). Future research should aim to study LGBTQ peopleand understand whether the risk factors for IPV/DV differs from that of heterosexual women. Also, the risk factors due to the unique culture, caste system, and geographical composition of Nepal do not represent the global context; hence the findings cannot be generalized.

## Implications for Research, Practice, and Policy

### Research

Most studies included in the systematic review focused on the combination of any type of violence; physical, sexual, and emotional. Where physical and sexual violence were studied, emotional violence appears to have been overlooked. Only six studies explicitly studied emotional violence. Therefore, more empirical research is needed to look at emotional violence and how this places males and females in both rural and urban areas at risk of other forms of violence. Likewise, some of the factors were identified as both risk and protective factors for VAW in the context of Nepal such as education level, decision-making capacity, mother-in-law, women holding patriarchal gender norms and attitude. Further studies are needed to assess whether these factors play a protective role in reducing VAW. Additionally, to substantiate the ecological framework, more qualitative research is needed to understand the in-depth interrelations between family, culture, community, and society to understand the scale and nature of VAW and girls.

### Practice

Although looking at the protective factors against violence was outside the scope of the systematic review, during the review process it was perceived that addressing the risk factors at individual and relationship levels could minimize the risk at the societal and community levels, as most of the risk factors were identified here. Implementing programs tailored to younger males to enable recognition of the damages DV has on both the perpetrators and the victims lives and the roles they can play to become more involved at identifying and intervening in VAW could minimize the risk for violence. For girls at school age, these programs could include building confidence in a way that would enable recognition of violence, and the ability to report violence and the ability to resist early or forced marriages. Moreover, school curriculums could be skill-oriented so that young women can become financially independent, therefore increasing their autonomy within families. Such interventions would go some way to reducing the prevalence of VAW in Nepal. The protective factors against violence are an important angle through which women’s risk to violence can be minimized. Recognizing different types of risk and protective factors and their impact on victims could benefit practice and service users.

### Policy

There have been several attempts at national and supranational levels to tackle VAW and girls in Nepal. However, there is a need for more concerted country-level action involving multisectoral interventions at various levels of the ecological framework, including homes, schools, healthcare settings, local communities, and wider society. Schools and workplaces could implement safeguarding policies that train professionals about recognizing the impact of DV and IPV on educational levels or workforce productivity. Laws and policies should be implemented intensely and could be coupled with other measures such as educational initiatives, heavy fines for early marriages, as well as the ability of victims to report violence in real-time and self-defense techniques from government and nongovernmental organizations. For an overview of research implications, see Table 3.

**Table 3.** Implications of Research, Practice, and Policy.

Area	Implications
Research	- More empirical research is needed to look at the emotional violence and how this places males and females in both rural and urban areas at risk of other forms of violence. - More research is needed to assess the factors that were identified as both risk and protective toward violence against women. - More qualitative research is needed to understand the in-depth interrelations between family, culture, community, and society to understand the scale and nature of violence against women and girls in context of Nepal.
Practice	- Recognizing different types of risk and protective factors and their impact on victims could benefit practice and service users.
Policy	- Laws and policies should be implemented intensely and should be coupled with other measures such as awareness building and educational initiatives. - Schools and workplaces should work on implementing safeguarding policies and training the professionals on recognizing the impact of domestic violence and intimate partner violence on education and workforce productivity.
